# Development and evaluation of two brief digital health promotion game booths utilizing augmented reality and motion detection to promote well-being at a gerontechnology summit in Hong Kong

**DOI:** 10.3389/fpubh.2022.923271

**Published:** 2022-09-23

**Authors:** Shirley Man-Man Sit, Agnes Yuen-Kwan Lai, Tai-on Kwok, Hoi-wa Wong, Yiu-lun Wong, Edward Chow, Yu-kwong Kwok, Man-Ping Wang, Sai-Yin Ho, Tai-Hing Lam

**Affiliations:** ^1^School of Public Health, The University of Hong Kong, Pokfulam, Hong Kong SAR, China; ^2^School of Nursing, The University of Hong Kong, Pokfulam, Hong Kong SAR, China; ^3^Technology-Enriched Learning Initiative, The University of Hong Kong, Pokfulam, Hong Kong SAR, China

**Keywords:** family happiness, health promotion, gerontechnology, digital technologies, augmented reality, Hong Kong, community event, information and communication technology

## Abstract

**Background:**

The acceleration of population aging calls for simple and effective interventions catered for older people. Gerontechnology, the combination of gerontology and technology, can promote quality of life in older adults. However, public health-related events incorporating information communication technology (ICT) for older people have seldom been evaluated.

**Objective:**

We reported the development and evaluation of two simple and brief digital health promotion games hosted at the annual Hong Kong Gerontech and Innovation Expo cum Summit (GIES) in 2018 and 2019 to promote well-being.

**Methods:**

Two game booths (Dinosaur Augmented Reality photo-taking in 2018, Sit-and-Stand fitness challenge in 2019) were designed by our interdisciplinary team. Four gaming technologies were employed: augmented reality, chroma key (green screen), motion detection and 3D modeling. Immediately after the game, we administered a brief questionnaire survey to assess participant satisfaction, happiness and perceived benefits, and collected qualitative data through observations and informal interviews.

**Results:**

Majority of 1,186 and 729 game booth participants in 2018 and 2019, respectively, were female (73.4% and 64.7%) and older adults (65.5 and 65.2%). Overall satisfaction toward the game booths was high (4.64 ± 0.60 and 4.54 ± 0.68 out of 5), with females and older adults reporting higher scores. Average personal and family happiness of participants in 2018 were 8.2 and 8.0 (out of 10). 90.3 and 18.4% of participants in 2019 chose one or more personal (e.g. enhance healthy living habits 62.4%, enhance personal happiness 61.6%) and family (e.g. enhance family happiness 15.6%, improve family relationships 10.8%) benefits of the game booth, respectively. Participants showed enthusiasm toward the technologies, and pride in their physical abilities in the fitness challenge.

**Conclusion:**

Our report on the development and evaluation of brief game interventions with ICT showed high satisfaction and immediate perceived benefits in community participants. Females and older adults reported higher satisfaction. Simple tools measuring happiness and perceived benefits showed positive results. Older adults were receptive and enthusiastic about the new technologies. Our findings can inform researchers and organizers of similar events. More research on simple and enjoyable ICT interventions is needed to attract older adults and promote their well-being.

**Trial registration:**

The research protocol was registered at the National Institutes of Health (Identifier number: NCT03960372) on May 23, 2019.

## Introduction

With population aging accelerating globally, promoting both physical and mental well-being in older people, with simple and effective interventions incorporating new technologies is urgently needed.

The Gerontech and Innovation Expo cum Summit (GIES) is an annual flagship event held in Hong Kong, co-hosted by The Hong Kong SAR Government and The Hong Kong Council of Social Service. It aims to promote the city's continuous efforts to develop innovative technology to tackle the major challenges presented by a rapidly aging society (1). Launched in 2017, the GIES invites local and overseas stakeholders to present technological applications and innovations over the four-day summit and exchange insights on how gerontechnology, the combination of gerontology and technology (2), can promote quality of life in the city's older population. The GIES is Hong Kong's largest gerontechnology public education event, and is held at the Hong Kong Convention and Exhibition Center, one of the city's largest exhibition venues, with about 50,000 annual attendees, 130 local and overseas exhibitors and 60 workshops and activities (3).

The Hong Kong Jockey Club SMART Family-Link (JCSFL) Project is a four-year, inter-disciplinary, cross-sectoral collaboration between academia and social services, initiated by the Hong Kong Jockey Club Charities Trust with a donation of over HK$157 million (US$20 million). Project partners include the School of Public Health (SPH) and Technology-Enriched Learning Initiative (TELI) at The University of Hong Kong (HKU), and 12 non-governmental organizations (NGOs), collaborating on the project's primary aim of advancing the use of information communication technology (ICT) in family services to enhance services and promote family well-being in Hong Kong. One of the five main JCSFL Project components is public education and advocacy campaigns to increase understanding of healthy family functioning. We previously conducted a process evaluation and shared our experiences from the JCSFL Project's first public education event, the one-day launch event for the public on 11 November 2018 that utilized ICT to promote family happiness (4). Fourteen digital game booths co-created by HKU and NGO partners utilizing different gaming technologies were hosted at the event with high satisfaction and positive feedback from both the participants and NGO social workers.

The JCSFL Project was first invited to join GIES in 2018, and again in 2019 because of positive responses and high interests from participants in the previous year. An exhibition game booth developed by HKU that utilized ICT to promote family well-being and sharing of happiness was hosted each year. The digital game in GIES 2018 was the Dinosaur Augmented Reality (DAR) booth using green screen technology and both motion and sound effects. Participants posed for photos with family members next to a virtual dinosaur on the screen while using different funny props. This was one of the games at the JCSFL Project launch event (4) with the addition of props.

The digital game in GIES 2019 was the “Sit-and-Stand” (SAS) booth using motion detection technology to test participants' physical fitness, developed according to the standard and widely implemented “30-Second Chair Stand Test” (5). Participants were challenged to complete as many sit-and-stands as possible in 30 seconds. The game could detect the correct posture of the participants and count the number of sit-and-stands. We added new technologies to the standard test so that it was more attractive to participants and passers-by, with the number of stands completed showing in real-time on a nearby screen. At the end of the game, participants could also see the results of previous challengers and gauge their own performances.

The GIES game booths had three objectives: (i) to promote family happiness, communication and well-being through ICT-assisted interventions; (ii) to assess participant satisfaction toward the game booths; and (iii) to promote the JCSFL Project to the public. Simple evaluation was conducted with participants immediately after the games.

Happiness is key to well-being and health, and is commonly defined as a composite of positive emotions, life satisfaction and coping resources (6), or a psychological balance and harmony (7). Happiness predicts desirable life outcomes in various domains (6), as happy individuals are successful in friendships, marriage, work performance and income, and health (8). Family happiness is fostered from family activities, where spending time with family members can promote positive family relationships and individual happiness (9). Positive family communication is crucial for a strong family system, and facilitates the sharing of values, feelings and ideas to maintain and enhance family well-being (10). More social interactions with family members and sharing happy feelings and experiences can stimulate and enhance family happiness.

For older adults, family is considered the most important source of social support (11) that influences happiness and perceived health (12, 13). Family happiness, interactions and relationships become increasingly more important in maintaining well-being in older adults, as social networks shrink and needs for support and caregiving increase (14–16). Receiving support from family can enhance older people's happiness, sense of security and self-confidence (15), and their social integration and participation in activities that promote health and healthy aging (17, 18).

Game satisfaction refers to the degree in which players feel gratified with their experience while playing a game (19). Satisfaction and enjoyment are two distinct yet positively correlated concepts (20) that influence the acceptance, use and success of games (19), and are often used interchangeably. Enjoyment can be generated from completing and mastering game challenges or tasks, as individuals respond to their own good performances with positive emotions, such as happiness and pride (21). In-game success predicts game enjoyment and mood repair, including increasing happiness and reducing negative emotions such as anger (22). High satisfaction and enjoyment would enhance players' motivation to play a game and the learning outcomes.

Our search of PubMed, Cochrane Library and ClinicalTrials.gov on 6 August 2022 using keywords of “health”, “technology”, “game”, “older adults” and “aging” yielded several reports of game-based interventions enhancing both physical and cognitive health in older adults (aged 60 and above) through improved balance (23–25), functional mobility (24–26), gait speed (24) and reduced risk of falls (23), as well as improved cognitive executive functions (27), reaction time (25) and processing speed (25, 28). A systematic review further identified exercise games as the most popular in game-based interventions for older adults (25). We found no existing trials or reports that described the development and evaluation of brief, ICT-supported digital games to promote both personal and family well-being in a community event, with the exception of our previous paper on process evaluation and experience sharing of digital games in a large community event (the launch event) (4).

The present paper reports the development and evaluation of two simple and brief digital health promotion games hosted at the GIES to promote well-being in Hong Kong. We hypothesized that participants of our brief games at GIES 2018 and 2019 would have high satisfaction toward the games and enhanced personal and family happiness, and high satisfaction would be associated with higher happiness scores and chosen perceived benefits of enhanced personal and family happiness.

## Methods

### Development

#### Game development and technologies

The games were conceptualized and developed by an interdisciplinary team at HKU, including computer engineers and public health researchers, based on two of the five JCSFL Project themes—SMART Communication (GIES 2018) and SMART Living Habits (GIES 2019) (4). The remaining three project themes are SMART Parenting, SMART Emotion and SMART Coping.

Four gaming technologies were used in the game booths, including augmented reality (AR, using technological devices to enrich the real environment with virtual objects and running in real-time), chroma key (green screen technique to layer images or video streams together based on color hues), motion detection (use of a sensor to detect specific movements) and 3D modeling (reconstructing three dimensional presentations of different objects). The duration of the games was designed to be 1–2 min to ensure short queue times. Gameplay revisions and feedback were discussed in regular meetings, with the DAR and SAS game booths finalized in October 2018 and 2019, respectively.

#### Dinosaur augmented reality game booth at GIES 2018

The DAR game was developed with the main goal of promoting family happiness and communication through taking fun family photos. A dinosaur element was included to add novelty and attract participants of different ages. Our Project had developed another interactive game with dinosaurs that was featured in our launch event (4).

The DAR game booth utilized three technologies including 3D modeling, chroma key, and AR. The booth setup consisted of a green screen and screen display, and participants could go inside the booth with their family members for photo-taking. We provided some funny props, which included toy swords, guns and bows and arrows, a feather duster, bamboo fan, shower brush and mosquito swatter. Participants were encouraged to choose any props, make funny poses and expressions, and the screen display allowed them to see the AR effects in real-time of them posing next to a virtual, moving dinosaur, along with sound effects. Photos and videos of participants were taken, and printed photos were given as a souvenir. Each photo had a unique QR code linked to a secure, online storage folder with all photos and videos from the booth to facilitate participants' sharing with family and friends to further promote family well-being.

Due to constraints of the size of the green screen, each session could only cater up to 6 participants. At least 3 staff members were present for a typical session to run the booth. Their tasks included inviting attendees to participate and explaining the game, running the game booth, taking photos and videos, and administering the post-game questionnaire using an iPad. In non-peak hours, one staff member would be sufficient to man the booths.

The DAR was previously featured and exhibited in the JCSFL Project's one-day launch event (4). Detailed information on the booths, technology used, and photos of participants engaging in the booths can be found in Supplementary Figure 1.

#### Sit-and-stand game booth at GIES 2019

The SAS game was developed with the main goal of promoting family happiness and healthy living habits through a fun fitness challenge. Development and design of the game was acccording to the “30-Second Chair Stand Test” (5). In older adults, this test is used to measure lower-limb muscle strength and assist with screening and risk assessment (29, 30). Normative values for the chair stand test in Hong Kong Chinese older adults aged 60–90 years and above range from an average of 12.3–7.9 for females, and 14–5.8 for males (29), with below average scores indicating a risk for falls. We used this as an opportunity for health promotion and education by emphasizing the importance of lower-limb muscle strength for activities of daily living and functional independence in older adults.

The SAS game utilized two technologies, including motion detection and AR. The motion-sensor captured limb movements, detected correct body postures and allowed for the automated calculation of number of sit-and-stands completed, while AR allowed for the game to be portrayed in a virtual living room setting. The game was developed into an iPad application and is available for free download by the public on the Apple App Store (https://apps.apple.com/app/id1522473762).

The booth setup consisted of an iPad (with the game application running) at the front to capture participants' movements and a corresponding screen display located behind them that showed the number of stands completed in real-time, with a countdown timer. Report cards showing participants' achievements were printed afterwards as a souvenir.

### Evaluation

#### Participants

The GIES is a public event that attracts participants of all ages, with activities and services mostly targeted at older adults and their families. Attendees include the general public (such as older adults, youth, carers), government officials, welfare sector practitioners and academics. The Hong Kong Jockey Club hosted several booths at GIES 2018 and 2019, held on 22–25 November 2018 and 21–24 November 2019, respectively, and the JCSFL Project was responsible for one booth (around 5 m^2^) each year.

#### Data collection

Participants were invited to answer a simple questionnaire immediately after playing the digital games on their satisfaction toward the game booths, happiness and perceived benefits. As many participants visited the booths with family members and friends, only one from each group was asked to complete the surveys to avoid long queues. HKU staff members assisted with survey administration *via* iPads. At the end of the survey, participants were also asked to voluntarily provide their contact information (email address or phone number) if they wanted to receive more information regarding the JCSFL Project and for any follow up evaluation. This information allowed us to build a database of contacts for future events and studies. Qualitative data were also collected *via* on-site observations and a few informal interviews with participants by HKU staff. Number of participant visits to the booths were also tracked and monitored in real-time based on records of survey submissions to assess traffic and help inform planning of future health promotion events and activities.

#### Measures

To avoid long queues and cater to an older population, we designed simple, one-item questions to assess participant satisfaction, happiness and perceived benefits. The whole process was designed to be quick (within 2 min) with minimal number of questions to enhance response rate and avoid long queues.

Participant satisfaction with the game booths was assessed by the question, “Are you satisfied with our booth today?” on a scale of 1 (not satisfied at all) to 5 (very satisfied) stars. This question was used in our previous launch event (4) and other community events with high response rates.

Willingness to practice sharing happiness from the game booths with family was assessed by the question, “After participating in our booth today, will you share the happy things or feelings from today's activity with your family?” with the answer options of “yes” or “no.”

For the 2018 event, two questions assessed personal and family happiness after participating in the DAR game, “Do you think you are happy?” and “Do you think your family is happy?” on a scale of 0 (very unhappy) to 10 (very happy). We used these questions in our previous studies (31–34), and have shown that the family happiness question is a reliable and valid tool (35).

For the 2019 event, we first designed a direct question on perceived benefits (PB) of the SAS game, “What benefits has this game brought you?” with a list of 9 answer options regarding (i) 4 personal benefits (enhanced personal happiness, enhanced healthy living habits, reduced stress, enhanced community connection), and (ii) 2 family benefits (enhanced family happiness, improved family relationships), as well as the option of choosing “other benefits” (with a “please specify” prompt to submit their own answers) and “no benefits at all”, plus a counterfactual option (increased monetary wealth). We used the counterfactual option to check against “social desirability bias” and haphazard answers, and expected null answers.

Simple demographic information of sex and age group (12 and below, 12–17, 18–29, 30–39, 40–49, 50–59, 60–69, 70 and above) was recorded for both events. Age groups were further categorized as children and teenagers (<12, 12–17), adults (18–59), and older adults (60 and above). In 2018, for brevity, staff confirmed the age groups of participants during survey administration (e.g., “Are you above the age of 60?”). In 2019, age was recorded based on participants' own choice of age group at the start of the SAS game (Supplementary Figure 1). Supplementary Figure 2 shows the two questionnaires used in GIES 2018 and 2019.

#### Statistical analysis

All statistical analyses were conducted using SPSS (version 26.0). All significance tests were two-sided with *P* < 0.05 indicating statistical significance. Independent *t*-test was used to compare satisfaction and happiness scores by age group and sex, and happiness scores in participants with low (1–3) and high (4–5) satisfaction. Chi-square test was used to compare differences in chosen perceived benefits of enhanced personal and family happiness in participants with low and high satisfaction.

## Results

### Participants

Our recruitment of GIES attendees to join our booths were quite successful, with about 80% of passers-by visiting our game booths. Attendees who visited our booths and completed the post-game questionnaire were counted as participants. Table 1 shows the majority of 1,186 and 729 game booth participants in 2018 and 2019, respectively, were female (73.4 and 64.7%) and older adults (65.5 and 65.2%). Very few were children/teenagers (6.1 and 0.7%).

**Table 1:** Participant characteristics of the Jockey Club SMART Family-Link (JCSFL) Project game booths at the Gerontech and Innovation Expo cum Summit (GIES) 2018 and 2019.

	**All^c^**	**2018**	**2019**
	**(*N =* 1,915)**	**(*N =* 1,186)**	**(*N =* 729)**
	***n* (%)**	***n* (%)**	***n* (%)**
**Sex**
Male	562 (29.3)	316 (26.6)	246 (33.7)
Female	1,342 (70.1)	870 (73.4)	472 (64.7)
**Age group** ^**a**^
Children and teenagers^b^	78 (4.4)	73 (6.2)	5 (0.7)
Adults	579 (30.2)	336 (28.3)	243 (34.1)
Older adults	1,241 (64.8)	777 (65.5)	464 (65.2)

Missing data excluded.

^a^Age intervals include children and teenagers (<12, 12–17), adults (18–59), and older adults (60 and above).

^b^Grouped together due to small numbers.

^c^GIES attendees that visited the game booths and completed the questionnaire survey.

**Figure 1:**
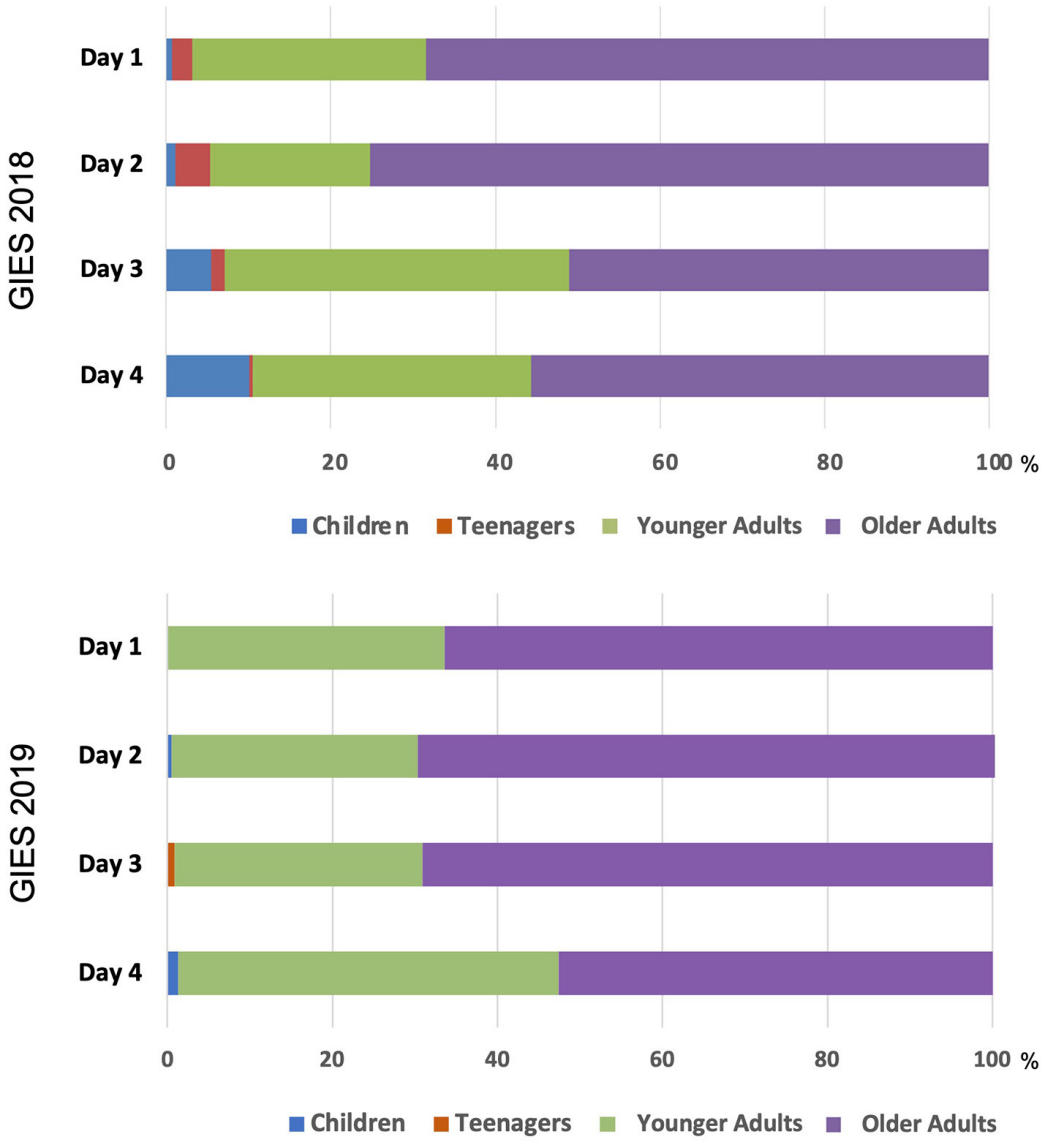
Age group of JCSFL Project game booth participants at GIES 2018 and 2019.

Figure 1 shows that the game booths in both years were visited most by older participants, with more older adults on weekdays than weekends. The proportions of children and teenagers, while small, were greater on weekends.

**Figure 2:**
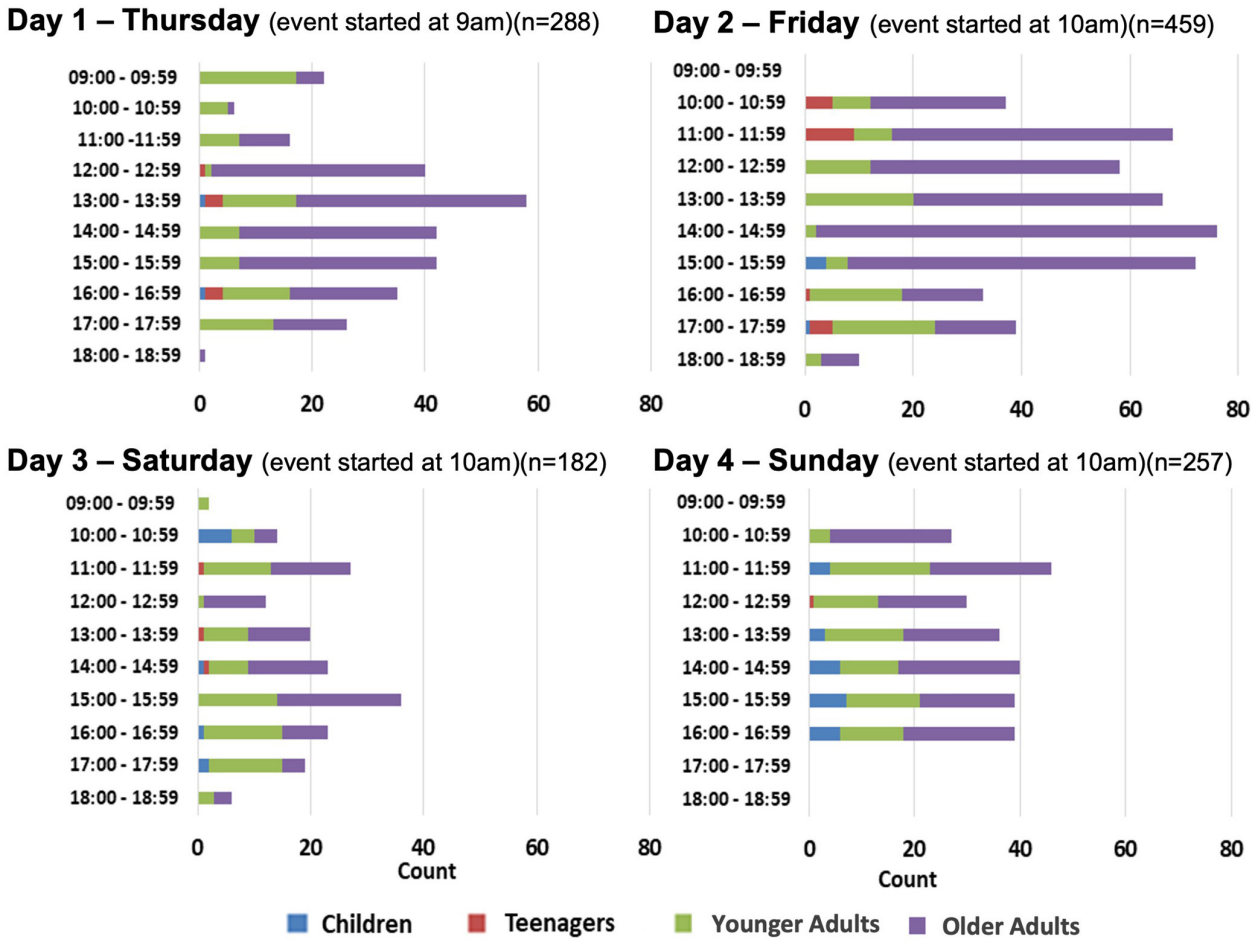
Number and age groups of JCSFL Project game booth participants by time at GIES 2018.

**Figure 3:**
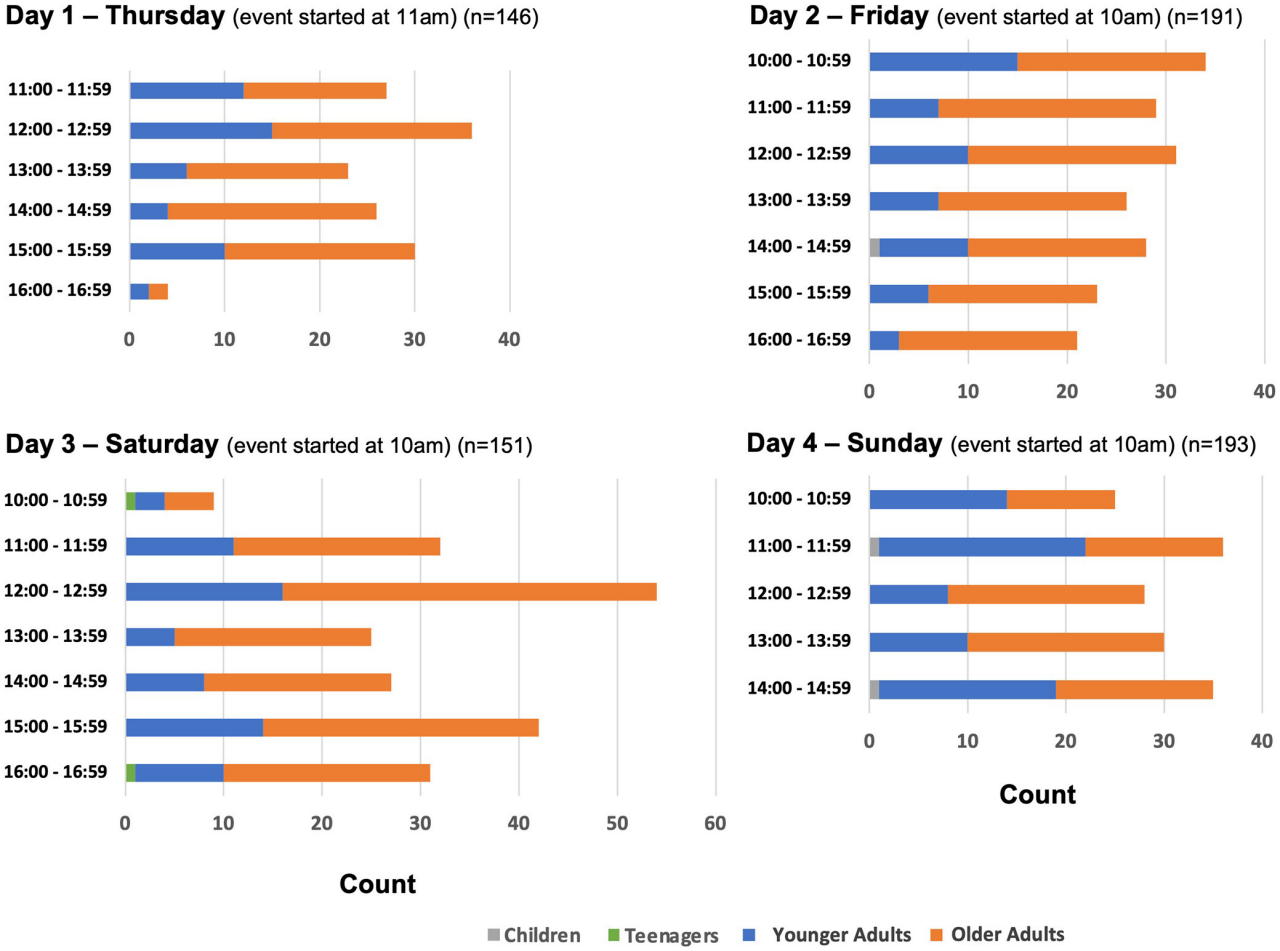
Number and age groups of JCSFL Project game booth participants by time at GIES 2019.

Figures 2, 3 show that the peak time of participants joining the game booths was between 11 am and 4 pm on Day 2 of GIES 2018, and between 12 pm and 4 pm on Day 3 of GIES 2019.

### Satisfaction toward game booths

Table 2 shows the average game booth satisfaction (±standard deviation) scores were 4.64 ± 0.60 and 4.54 ± 0.68 for 2018 and 2019, respectively, with 94.7 and 92.0% of participants giving satisfaction scores of 4 or 5. Females and older adults had significantly higher satisfaction scores than their respective counterparts for the 2 years combined (*P* = 0.002 and *P* = 0.001) and for 2018 (both *P* = 0.002). Almost all participants (96.9 and 94.1%) reported their willingness to share happy things or feelings from the activities with family members.

**Table 2:** Satisfaction toward JCSFL Project game booths at GIES 2018 and 2019.

	**All**		**2018**		**2019**		***P***
	***N***	**Score** **(mean ±SD)**	***N***	**Score** **(mean ±SD)**	***N***	**Score** **(mean ±SD)**	
Overall	1,915	4.60 ± 0.63	1,186	4.64 ± 0.60	729	4.54 ± 0.68	0.002
**Sex**
Male	562	4.53 ± 0.67	316	4.54 ± 0.66	246	4.51 ± 0.69	
Female	1,342	4.63 ± 0.62	870	4.67 ± 0.57	472	4.55 ± 0.68	
		*P =* 0.002		*P =* 0.002		*P =* 0.40	
**Age group**
Children/teenagers/younger adults	654	4.53 ± 0.71	407	4.56 ± 0.70	247	4.49 ± 0.72	
Older adults	1,238	4.64 ± 0.58	775	4.68 ± 0.53	463	4.58 ± 0.73	
		*P =* 0.001		*P =* 0.002		*P =* 0.08	
Satisfaction score of 4 or 5 (%)	1,812	94.6	1,122	94.7	690	92.0	0.021
Willing to share happy things or feelings from activities with family (%)	1,843	95.9	1,137	96.9	706	94.1	0.003

Satisfaction score on a scale of 1 (not satisfied at all) to 5 (very satisfied).

### Personal and family happiness at GIES 2018

**Figure 4:**
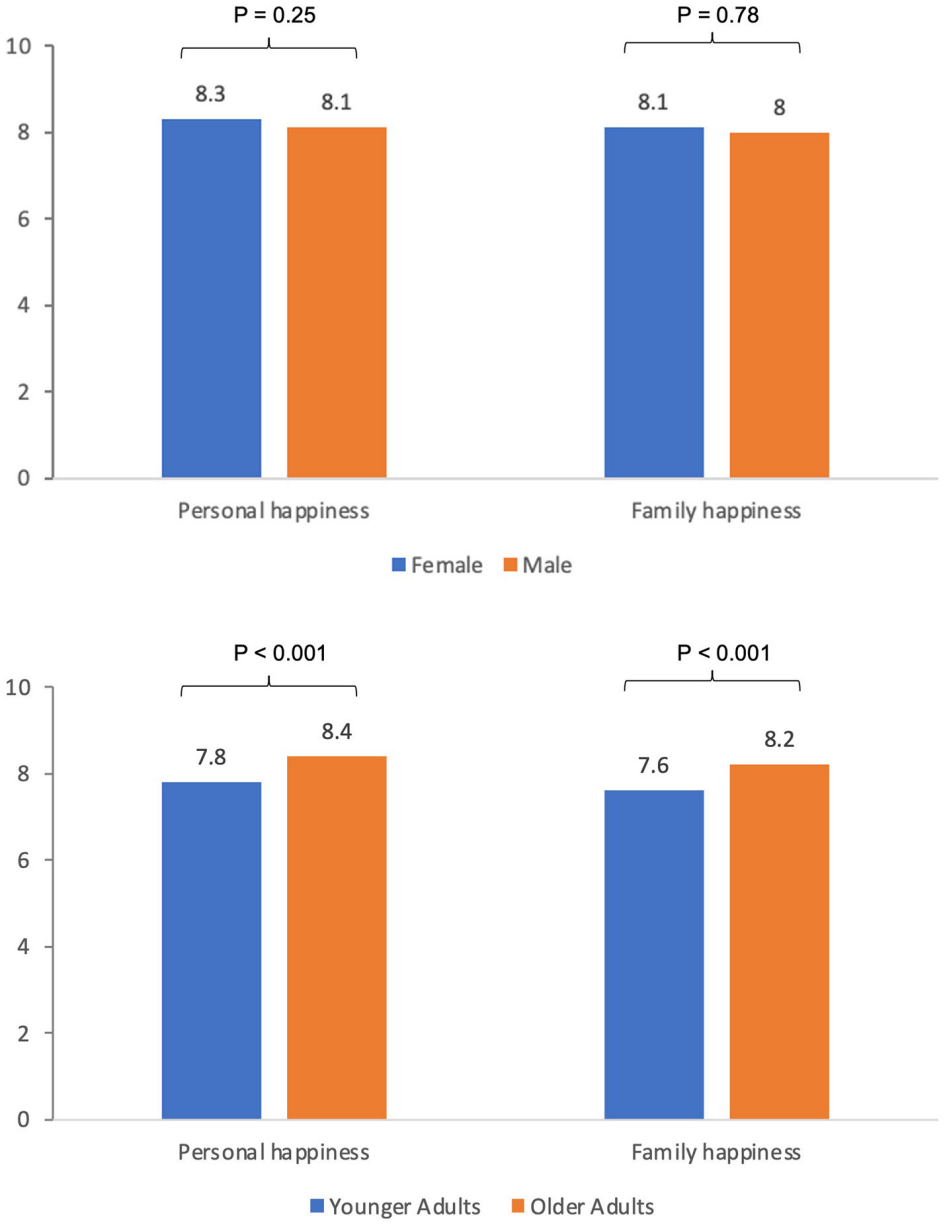
Personal and family happiness scores of JCSFL Project game booth participants at GIES 2018.

The average scores of personal and family happiness of participants were 8.2 and 8.0 (out of 10), respectively. Figure 4 shows younger adults had significantly lower scores for both personal and family happiness than those of older adults (both *P* < 0.001). No significant differences in personal (*P* = 0.25) and family happiness (*P* = 0.78) scores were observed between females and males.

### Perceived benefits at GIES 2019

Table 3 shows the most chosen personal perceived benefit of the activity at GIES 2019 was enhanced healthy living habits (62.8%), followed by enhanced personal happiness (61.6%) and reduced stress (35.5%). The most chosen family perceived benefit of the activity was enhanced family happiness (15.6%). Other chosen perceived benefits included enhanced balance, increased exercise and increased knowledge of the JCSFL Project. 90.3 and 18.4% chose one or more personal and family benefits, respectively. 2.9% chose the counterfactual option of “increased monetary wealth”—our staff asked a follow-up question to those who chose this option, and the overall replies were: “enhanced happiness and health mean enhanced monetary wealth”.

**Table 3:** Perceived benefits from JCSFL Project game booth at GIES 2019 (*N* = 718).

		**Sex**			**Age group** ^**c**^		
	**All**	**Male**	**Female**	***P***	**Younger adults**	**Older adults**	***P***
	**(*N =* 718)**	***N =* 246**	***N =* 472**		***N =* 243**	***N =* 464**	
	**%**	**%**	**%**		**%**	**%**	
**Personal benefits**
Enhance healthy living habits	62.4	61.0	62.5	0.69	62.6	62.1	0.71
Enhance personal happiness	61.6	61.0	62.5	0.92	59.7	62.7	0.51
Reduce stress	35.5	40.2	33.3	0.06	36.6	34.5	0.83
Enhance community connection	15.5	16.3	14.8	0.61	15.6	15.9	0.62
Increase monetary wealth^a^	2.9	3.7	2.5	0.40	2.5	3.2	0.79
Any personal benefit	90.3	90.7	90.0	0.79	91.4	89.7	0.59
**Family benefits**
Enhance family happiness	15.6	17.9	14.2	0.19	14.4	16.8	0.44
Improve family relationships	10.8	13.4	9.1	0.08	8.2	12.3	0.19
Any family benefit	18.4	20.3	17.4	0.33	16.0	20.3	0.22
Other benefits^b^	7.0	4.1	8.7	0.02	7.0	6.9	0.83

^a^Counterfactual answer option.

^b^Examples of benefits submitted by participants include: gained new knowledge, new experience, able to learn about the JCSFL project.

^c^Children and teenagers were excluded due to small numbers.

### Satisfaction toward game booths and enhanced happiness

Table 4 shows participants who gave high satisfaction scores of 4 or 5 reported higher personal and family happiness scores (both *P* < 0.001) at GIES 2018. Table 5 shows no differences between satisfaction and choosing perceived benefits of enhanced personal (*P* = 0.11) or family happiness (*P* = 0.68)”.

**Table 4:** Game satisfaction and happiness scores at GIES 2018.

	**Personal happiness**			**Family happiness**		
	***N***	**Score (0–10)**	***P***	***N***	**(0–10)**	***P***
**Satisfaction score**			< 0.001			< 0.001
1–3	63	6.76 ± 1.73		63	6.62 ± 1.85	
4–5	1,119	8.32 ± 1.68		1,109	8.11 ± 1.96	

Satisfaction score on a scale of 1 (not satisfied at all) to 5 (very satisfied).

Personal and family happiness on a scale of 0 (very unhappy) to 10 (very happy).

**Table 5:** Game satisfaction and perceived benefits of enhanced personal and family happiness at GIES 2019.

	**Satisfaction score**		
	**(*N = 727*)**		
	**1–3 (%)**	**4–5 (%)**	***P***
**Enhance personal happiness**			0.11
Yes	4.1	57.5	
No	3.9	34.5	
**Enhance family happiness**			0.68
Yes	1.1	14.6	
No	6.9	77.4	

Satisfaction score on a scale of 1 (not satisfied at all) to 5 (very satisfied).

### Qualitative evaluation *via* on-site observations

For both years, our staff made on-site observations of participants and conducted a few informal interviews with them. At GIES 2018, 1,470 photos and videos of participants were taken. These photos, as well as on-site observations of the partcipants' behaviors and responses, showed that they were happy and laughing, and had a fun time choosing props for the photo-taking. Many also expressed their interests and amazement toward the AR technology, as they had never experienced it before. They particularly liked the photos with the dinosaur, and some forwarded the photos immediately to their family members and friends *via* WhatsApp.

At GIES 2019, participants were excited for the opportunity to challenge themselves and test their physical fitness. Many older participants expressed surprise at their physical ability as shown by the high number of sit-and-stands and were very proud of themselves. Some participants asked to try the challenge again, and a few also revisited the booth with family and friends to participate together.

### Community connection

559 (47.1%) and 396 (54.3%) participants from each year agreed to leave their contact information (phone number or email address) to receive more information regarding the project and promoting family happiness. A total of 726 phone numbers and 310 email addresses were collected from both years and added to our project participant database. We were able to send them updated information about our project, related community events, and materials to promote family well-being, and build social and community connections.

## Discussion

We reported the development and evaluation of two very brief (<2-min) game interventions with ICT (the Dinosaur Augmented Reality (DAR) and Sit-and-Stand (SAS) game booths) that showed high satisfaction, and the SAS game additionally showed immediate perceived benefits in participants of different age groups. Females and older adults reported higher satisfaction. Participants of the DAR booth reported personal and family happiness scores of 8.0 and 8.2 out of 10, respectively, and participants of the SAS booth perceived various personal and family benefits.

Higher satisfaction in our female participants than males is consistent with previous literature showing females were more engaged and receptive toward technology-based interventions and devices (36, 37). While younger people are more accustomed to using a greater breadth of different technologies, recent surveys have shown increased use of technology in the older population with different usage patterns and frequencies compared with younger people (38, 39). Despite older people being more inclined to use more old-fashioned technologies, which suggests a lag in access and usage of more recent technologies (39), our results showed older adults were very interested in our games and new technologies. Our on-site observations also corroborated this, as older participants showed great enjoyment and were very receptive and enthusiastic. Many stayed behind to ask questions about the game design and technologies used. Although direct comparison might be limited by different methods and contexts, satisfaction toward our game booths appeared high (4.64 and 4.54 out of 5), with reference to satisfaction scores reported in other exercise game interventions for older adults [3.45 out of 5 (40) and 49.2 out of 56 (41)].

Interventions utilizing technology have been catered primarily for children and younger adults, who are considered more receptive (42). Alternatively, our results can inform researchers and organizers of similar events on the value of such interventions for older adults. Older adults tend to have less exposure to games with new technologies such as AR and motion detection, and may be more easily satisfied with such experiences, even when the games are very brief and simple. Younger participants, who more likely have had previous experiences, may have higher expectations and like something more challenging. Developing games with tasks or challenges that older adults can complete, while providing appropriate support and guidance to facilitate a positive experience, can increase game enjoyment and satisfaction, and promote positive emotions such as happiness and increase self-esteem (21). Our results showed that participants at GIES 2018 who gave higher satisfaction scores also reported higher personal and family happiness. This suggests the great potential and need for the development and integration of simple, ICT-assisted interventions to promote happiness and well-being in older adults, and indicates that such interventions would be received positively.

Older adults have specific needs for the design of interactive games and exercise technologies, including instructions (providing demonstrations of exercise/activities and personalized feedback) and accessibility (providing detailed instructions on setup of technology, and continuous instructions/reminders). Their involvement in the design and development process is therefore crucial to the acceptability and success of such games and should be carefully considered (43, 44).

Our game booths had some strengths. First, the interventions were simple and brief, incorporating new and attractive technologies that older participants were less likely to have had prior experiences with, such as AR. The use of AR has been increasing in fields of retail, medicine and entertainment, including multi-player gaming and computer games (45). AR can provide a more immersive and fulfilling experience for how people interact and engage in different activities, and allow for personalization and enhanced interactivity. Research has also shown that exercise games, such as our SAS booth, have higher enjoyment and promote sustainable physical activity over traditional interventions (46, 47).

Second, we were also able to use simple, straightforward tools to evaluate participant feedback using two different approaches, including measuring participants” personal and family happiness after participating in the DAR game booth, and participants” perceived benefits of the SAS game. These two approaches allowed us to gain insight into the immediate effects of the interventions, which were positive and encouraging. We are the first to report a very brief intervention resulted in several immediate perceived personal and family benefits, although these were self-reported with uncertain longer-term outcomes. More than 90% of participants who visited the SAS booth chose at least one personal benefit, with almost two-thirds choosing enhanced healthy living habits. Surveys have reported older people showing high interest and enthusiasm toward trying and adopting new technologies, especially if they perceive benefits and value from usage (48, 49). All participants answered all our questions with no problems or queries. The inclusion of a counter-factual answer option that <3% participants chose, and not all benefits were chosen, suggested that social desirability bias, if any, was not substantial.

Within the constraints of a brief intervention and simple evaluation, to be able to show high satisfaction and self-reported benefits cannot be found in similar interventions in the literature. The positive results and experiences from using these evaluation questions also subsequently guided our studies and evaluations for ensuing Project events and surveys, including the perceived benefits and harms of the COVID-19 pandemic (34, 50–52).

Another strength was that we had deployed substantial manpower (at least 6 people) to run each booth. Common barriers to technology use and adoption among older adults include lack of clarity in instructions and support, and fear of incorrectly using technology (53, 54). We had staff members on-site, inviting and explaining the games to participants with patience, encouragement and cheers. Such support made them feel more confident and comfortable, and they overcame such barriers. The additional manpower was also to facilitate health promotion on the benefits of family activities and exercise, and we strived to empower and encourage older adults to do more exercise at home by themselves and with family members. Many participants also revisited the booths with their family members. Intergenerational support in families can also help older adults overcome barriers in technology use and adoption (55), and help improve family relationships and social connectedness (56).

Through our game booths, we also explored two types of user experiences of ICT interventions, which could be categorized into “active” and “passive”; active users have control over the technology, whereas passive users do not (57). The DAR booth allowed participants to be fully immersed in a different environment (i.e., posing next to a dinosaur). In the SAS booth, participants completed the fitness challenge whilst facing away from the screen that displayed the automated counter and AR elements, so the technology integration was primarily enjoyed by their family members and passers-by. Participants saw the number of completed sit-and-stands on screen after completion. They received a paper record of what they achieved so that they could show others and share the happiness with family members and friends. Satisfaction toward technology could be impacted by how much control a user has with the technology (57), so investigating differences in satisfaction and benefits of ICT interventions with active or passive user experiences is warranted.

Many game booths have been setup in public health-related events in Hong Kong and elsewhere with different formats, aims and uses of ICT, and requiring much effort and resources for implementation. However, these events mostly involved no quantitative or qualitative evaluation, other than observing responses of participants. The development, evaluation and success of our brief ICT interventions can serve as a useful reference for organizers of similar events and their evaluations, and our results suggest brief and innovative interventions with ICT can be successfully integrated to promote well-being in different age groups and communities. Further research on longer term effects is warranted. Based on our experiences from the GIES, we hosted additional game booths under JCSFL Project in other health promotion settings, in which details may be reported in the future. Our Project has also developed over a dozen game applications (in Chinese only) to promote family-related themes such as parenting, communication and coping that can be downloaded for free by the public (https://jcsmartfamilylink.hk/appindex/). Evaluations on user satisfaction and perceived benefits of these applications may also be reported in the future.

Hong Kong has continuously had the highest life expectancy globally for the past seven years (58), and thus has maintained a strong emphasis on finding solutions to improve quality of life for older adults. The GIES was launched in 2017 as a knowledge transfer platform for stakeholders to exchange ideas and collaborate on adoption of gerontech, and drive policy change to help the city cope with a rapidly aging population. Gerontechnology has been successful in helping older adults live healthier and more independently (59, 60). However, disparities in technology use and penetration in Hong Kong remain a concern. A 2020 local survey found almost all adults aged 25 to 44 reported internet usage (99.8%) and owned smartphones (99.7%), compared with 65.9% and 68.1% in adults aged 65 and older, respectively (61). Thus, more events and activities like the GIES catering to older adults should be held to allow exposure and provide opportunities to build confidence and comfort in technology use.

Our study had some limitations. First, no follow-up evaluation was conducted after the event, as we expected very few participants would consent. Second, our perceived benefit questions had only yes/no answer options but no scores. Third, only about 10% of GIES attendees visited our game booths, and fewer completed questionnaires. There were many other booths with exhibition and games, and the venue was very large. Generalizability of results are therefore uncertain. There were also fewer GIES attendees in 2019 due to social unrest in the city, which attributed to a smaller sample size. Fourth, social desirability bias, though unlikely to be substantial, could not be ruled out completely. Fifth, no validated tools were used for measuring perceived benefits, but the specificity in responses to our one-item question and the inclusion of a counter-factual answer option that <3% participants had chosen supported the validity of the data. Lastly, older adults included in our results were those able to attend the GIES, either by themselves or assisted by others, therefore generalizability may be limited. Interventions that can reach other older adults are needed.

## Conclusion

Our report on the development and evaluation of brief game interventions with ICT showed high satisfaction and immediate perceived benefits in community participants. Females and older adults reported higher satisfaction. Older adults were receptive and enthusiastic about new technologies. Simple tools measuring happiness and perceived benefits showed positive results. More research on simple ICT interventions is needed to attract older adults and promote their well-being.

## Data availability statement

The datasets presented in this article are not readily available because the sharing of data to third parties was not mentioned in the consent form for participants. Requests to access the datasets should be directed to SH, syho@hku.hk.

## Ethics statement

The studies involving human participants were reviewed and approved by the Institutional Review Board of the University of Hong Kong/Hospital Authority Hong Kong West Cluster (IRB Reference No.: UW19-247). The patients/participants provided their written informed consent to participate in this study.

## Author contributions

AL, Y-kK, M-PW, S-YH, and T-HL contributed to the conception and design of the study. SS, T-oK, H-wW, Y-lW, and EC contributed to the implementation of the program. SS wrote the first draft of the manuscript. SS and AL analyzed the data. SS, AL, S-YH, and T-HL critically reviewed and revised the manuscript. All authors read and approved the final manuscript.

## Funding

The research was funded by the Hong Kong Jockey Club Charities Trust.

## Conflict of interest

The authors declare that the research was conducted in the absence of any commercial or financial relationships that could be construed as a potential conflict of interest.

## Publisher's note

All claims expressed in this article are solely those of the authors and do not necessarily represent those of their affiliated organizations, or those of the publisher, the editors and the reviewers. Any product that may be evaluated in this article, or claim that may be made by its manufacturer, is not guaranteed or endorsed by the publisher.
