# Supplementary material for: Development and evaluation of two brief digital health promotion game booths utilizing augmented reality and motion detection to promote well-being at a gerontechnology summit in Hong Kong
Source: Front Public Health. 2022 Sep 23;10:923271. doi: 10.3389/fpubh.2022.923271 (PMC9539530; doi:10.3389/fpubh.2022.923271)
Supplement: Supplementary file 1 [file Data_Sheet_1.PDF]

Supplementary Figure 1. Descriptions, equipment lists and pictures of game booths

<u>Dinosaur Augmented Reality (DAR) Game Booth at GIES 2018</u>

"Promoting personal and family happiness and communication by taking fun family photos and posing next to a virtual dinosaur"

Technology used:

- 3D Modelling
- Chroma Key
- Augmented Reality

Equipment list:

- Screen display
- Computer [with game program pre-loaded]
- Shooting supplies [includes 4K camera, tripod and green screen]
- Props [different toys and household items such as dusting mop, toy sword and toy gun]

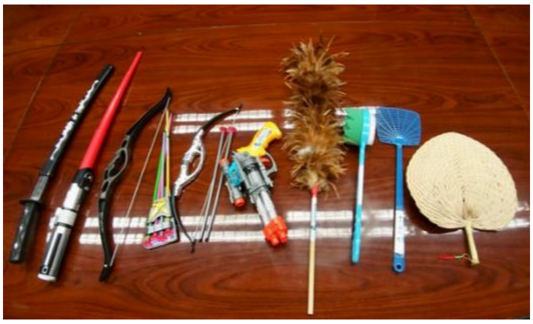

Props used for the DAR game booth

User experience:

Participants choose different props and pose in front of a green screen and take a family photo next to a virtual, moving dinosaur. They are encouraged to do funny poses, and a screen display is placed in front so they can see the AR effects. Photos are printed and given to participants as a souvenir.

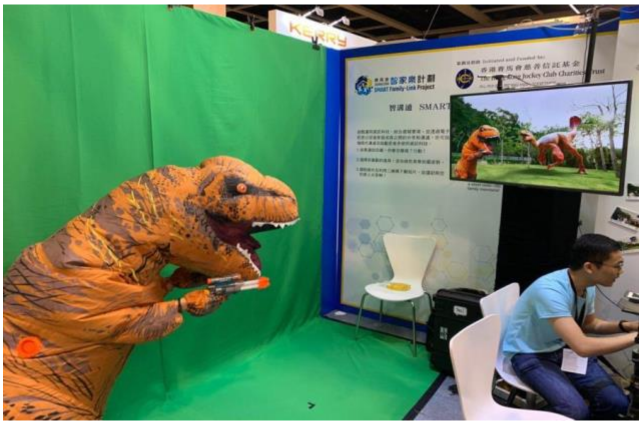

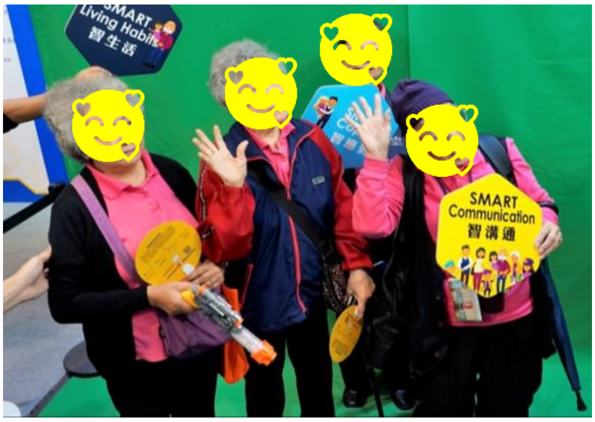

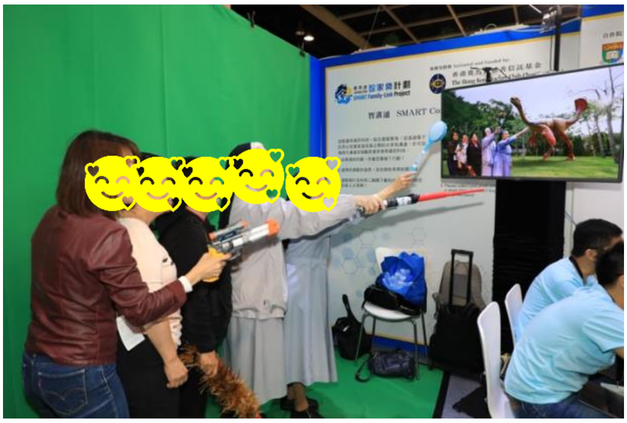

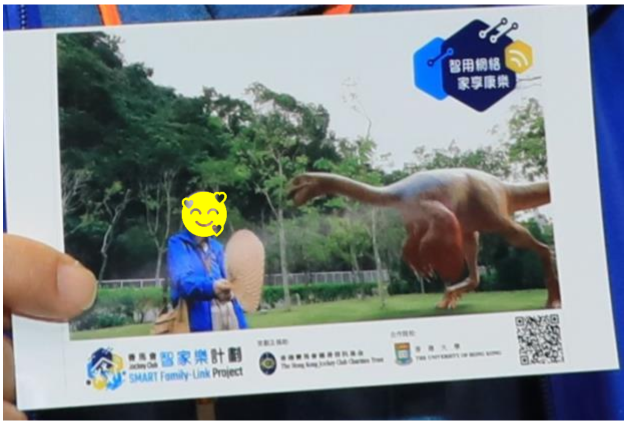

<u>**Sit and Stand (SAS) Game Booth at GIES 2019**</u>

"Promoting physical activity and healthy living with family members through a timed sit-and-stand fitness challenge"

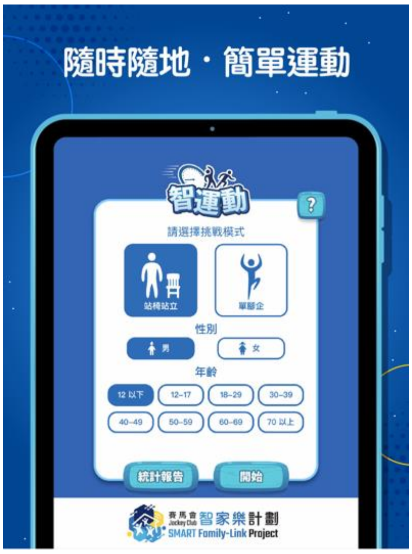

Technology used:

Motion Detection
Augmented Reality

Equipment list:

Screen display
iPad [with game program pre-loaded*]
iPad stand
Chair
*game can be downloaded for iPad on the App Store:
https://apps.apple.com/app/id1522473762

User experience:

Participants take turns in this fitness challenge and complete as many sit-and-stands as possible in 30 seconds using the chair provided. The iPad program captures the number of sit-and-stands and shows the elapsed time. A report card with their achievements is printed and given to each participant as a souvenir.

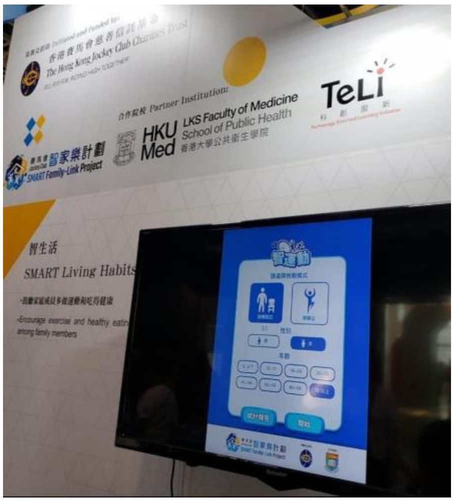

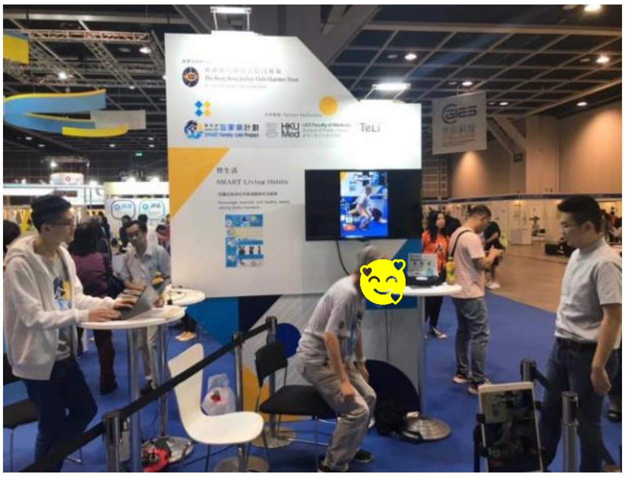

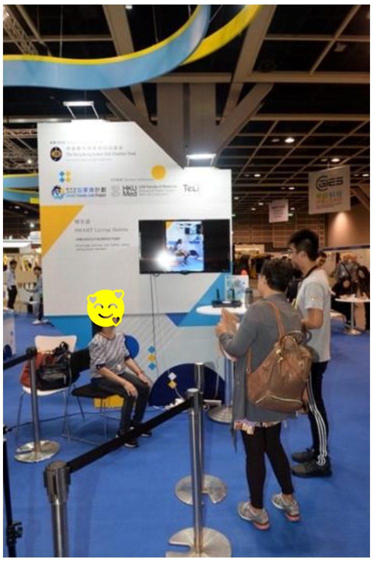

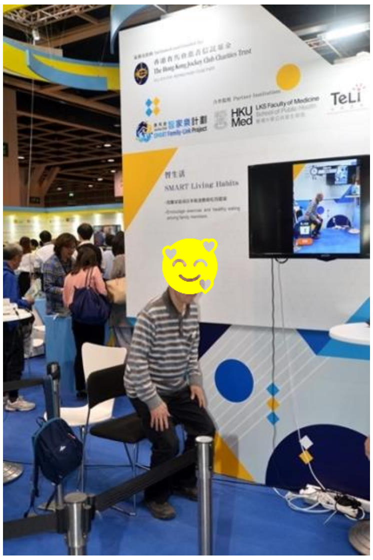

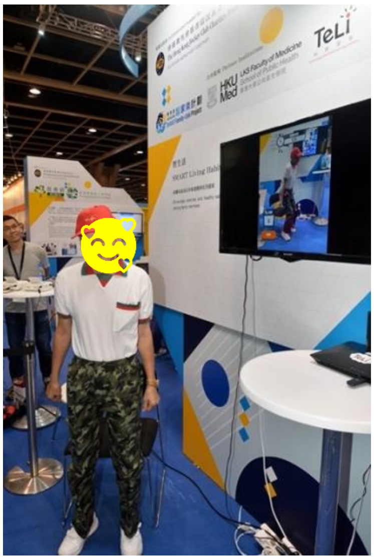

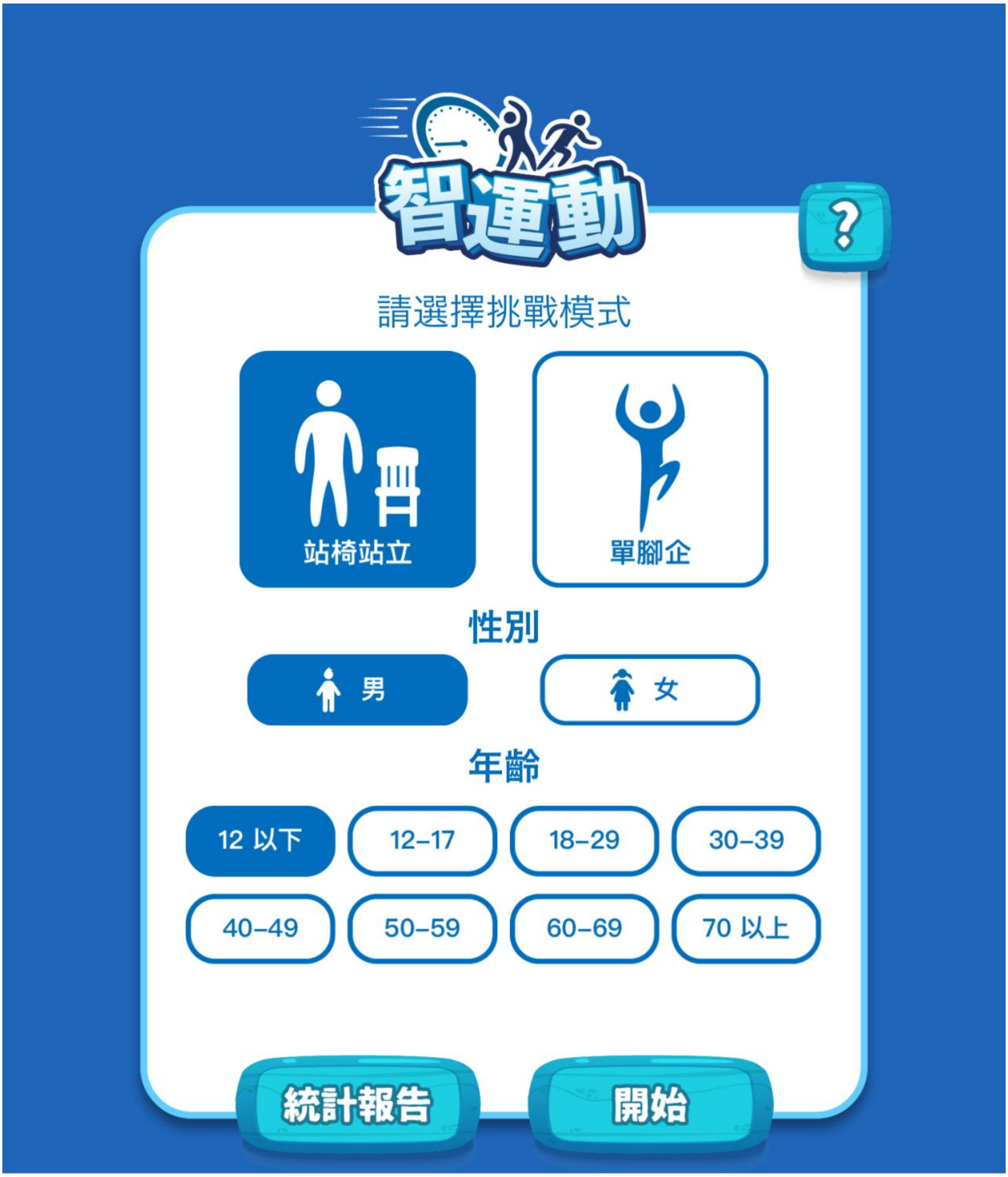

賽馬會 Jockey Club 智家樂計劃
SMART Family-Link Project

Supplementary Figure 2. Questionnaire for GIES 2018 and 2019

樂齡科技博覽暨高峰會 2018
活動後問卷

以下問題是有關本活動的整體評價，請於活動後填寫並選出最能代表你的答案。收集的數據只作學術研究及統計之用，個人私隱絕對保密。

		非常不滿意				非常滿意
1.	你對今天的「智溝通」攤位滿意嗎？	☆	☆	☆	☆	☆
					會	不會
2.	參加了今天的「智溝通」攤位後，你會不會與家人分享今天活動中快樂的事或感受？				☐	☐
3.	你覺得自己快樂嗎？				0 = 非常不快樂	10 = 非常快樂
4.	你覺得你的家庭快樂嗎？					

你願意留下聯絡電話或電郵以收取有關「賽馬會智家樂計劃」的資訊和日後的跟進？如願意, 請填:
姓氏: ____________________________
電郵: ____________________________
電話: ____________________________

個人資料

性別:	☐ 男 ☐ 女			
年齡:	☐ 小童	☐ 青少年	☐ 成人	☐ 長者

樂齡科技博覽暨高峰會 2019
活動後問卷

以下問題是有關本活動的整體評價，請於活動後填寫並選出最能代表你的答案。收集的數據只作學術研究及統計之用，個人私隱絕對保密。

		非常不滿意				非常滿意
1.	你對今天的「智運動」攤位滿意嗎？	☆	☆	☆	☆	☆
					會	不會
2.	參加了今天的「智運動」攤位後，你會不會與家人分享今天活動中快樂的事或感受？				☐	☐

3. 本活動有給你帶來以下得益嗎? (可選多項)

① 增加個人快樂	② 提高健康生活意識	③ 減少壓力	④ 增加社區聯繫	⑤ 改善家庭關係
⑥ 改善家庭關係	⑦ 增加金錢財富	⑧ 其他得益 (請說明:____)	⑨ 完全沒有	

你願意留下聯絡電話或電郵以收取有關「賽馬會智家樂計劃」的資訊和日後的跟進？如願意, 請填:

姓氏: ____________________________

電郵: ____________________________

電話: ____________________________

個人資料

性別:	☐ 男 ☐ 女			
年齡:	☐ 小童	☐ 青少年	☐ 成人	☐ 長者
